# A study on the types of disaster awareness in nursing students: Q methodology

**DOI:** 10.1186/s12912-023-01636-8

**Published:** 2023-12-08

**Authors:** Mihyeon Seong, Dajung Ryu, Sohyune Sok

**Affiliations:** 1 DKMediinfo Nursing Information Research Institute, Changwon-si, Gyeongsangnam-do Republic of Korea; 2 Department of Nursing, Graduate School, Kyung Hee University, Seoul, Republic of Korea; 3 College of Nursing Science, Kyung Hee University, 26, Kyungheedae-ro, Dongdaemun-gu, Seoul, 02447 Republic of Korea

**Keywords:** Nursing student, Disaster, Safety, Q methodology

## Abstract

**Background:**

Research for the development of nursing education strategies to enhance the competency of the nursing students on disaster safety are needed. This study aimed to identify the types of perceptions on disaster safety in nursing students, and to analyze and describe the characteristics of each type of disaster safety perception of nursing students in South Korea.

**Methods:**

An exploratory study design applying Q methodology, a research method designed to study subjectivity. Participants were 30 nursing students in their 20s who are living in C city. This P-set was selected to best reveal the disaster safety awareness of nursing students. Participants provided their subjective viewpoints by sorting 30 statements into a grid. Analyses involved correlation and factor analysis. The study was carried out from June to December, 2020.

**Results:**

In this study, four types of disaster safety awareness of nursing students were uncovered. The characteristics of each type were confirmed as follows: Type 1 was national responsibility, type 2 was individual responsibility, type 3 was preparedness-oriented, and type 4 was education-oriented.

**Conclusion:**

This study shows that the types of perceptions on disaster safety in Korean nursing students were national responsibility, individual responsibility, preparedness-oriented, and education-oriented. The findings from this study can be implied as fundamental data in nursing education of disaster safety.

## Background

Disaster is an event that occurs when demand exceeds immediately available resources [1, 2]. It can also be defined as an event that overwhelms local capacity and requires a request from external resources at the national or international level [3]. Disasters occur frequently all over the world, and large-scale disaster events are constantly occurring in South Korea. Furthermore, the number of occurrences is increasing every year [4].

Nurses provide nursing care relevant to each disaster stage by helping those who cannot solve their own health problems in an environment impacted by the disaster [5]. Before a disaster occurs, it is necessary to identify and prepare for individual-level and community-level problems, and establish a plan to save as many lives as possible. Nurses are important health care workers in disasters, as they are responsible for identifying additional needs of people and play a pivotal role in all stages of a disaster [6].

Due to the severity of disasters that are increasing around the world, the role of nurses to patients in disasters is being emphasized, and the demand for improving the ability of nurses to respond to disasters is increasing [7, 8]. Nursing capacity, which requires nurses to have knowledge and skills that are unique to disasters, is also required for nurses providing care during a disaster when they may have scarce resources and work in suboptimal environments during disaster situations [9–11]. Unlike clinical trials with well-equipped resources and well-trained nurses, most disaster sites are in suboptimal environments that require a response with limited resources and often in the absence of advanced systematic disaster safety preparedness education.

Labrague et al. [7] reported that disaster response capabilities of nurses were closely related to previous disaster nursing experience or disaster-related education. However in particular, nursing students, who will become nurses in the future, lack awareness of disasters and disaster nursing, and they do not fully understand the required role of nurses in disasters [12]. For this reason, it is essential for nurses to acquire knowledge and skills in order to respond promptly to disaster situations. The need to provide the basics for disaster nursing to nursing students has also been raised [12, 13]. Disaster education should be included as an essential part of the nursing curriculum because all disasters start locally, and nurses must be aware of the various disaster situations that can occur, as well as have adequate and effective response capabilities [14].

Nevertheless, disaster education is covered only in some nursing schools, and research on disasters knowledge among nursing students is insufficient [14–16]. Nurses or nursing students in Korea have a vague conception of disaster or disaster nursing and may fall into a panic when an actual disaster occurs [15].

At a time when various data are needed to organize a systematic disaster preparedness curriculum, it is necessary to develop disaster awareness through systematic and appropriate training in undergraduate courses in order to respond with sufficient capacity to sudden disaster situations [9, 17]. According to a study by Woo, et al. [18], it is argued that disaster awareness should be started from undergraduate courses because disaster awareness is correlated with disaster preparedness. Also, according to Kwon, et al. [19], it was suggested that nursing students should increase their awareness of disasters and cultivate their ability to prepare and cope with disasters through disaster education. This disaster education is necessary for nursing students to be aware of the paradigm that reflects social and medical changes, to prepare for the future society, and to establish a value system for humanities and nursing professions in the undergraduate course of the university in order to develop the ability to cope with various problem situations to be faced in the nursing field [20]. It is important to first explore the perception of disaster safety recognized by nurses who will work in the nursing field in the future.

Therefore, this study seeks to understand the disaster safety as it is perceived by nursing students. The study uses Q methodology to reveal subjective perspectives about disaster safety from the perspective of nursing students.

The purpose of this study was to uncover the types of subjectivities held by nursing students relative to disasters and to describe each type of perspective. The results will provide the fundamental data necessary for research and development of nursing education strategies that would be effective in cultivating nurses who could prepare for various disaster situations by confirming the diverse perspectives of the nursing students and enhancing their understanding of disaster. Accordingly, the aims of this study were to identify the types of awareness of disaster in nursing students, and to analyze and describe the characteristics of each type of disaster awareness of nursing students.

## Methods

### Study design

This study is an exploratory study that applied Q methodology, a subjective research method, to explore disaster safety awareness of nursing students more systematically and scientifically. Q methodology is a methodology that uncovers and describes the multiple viewpoints about a topic or event. That is, it is a way to find correlations between people across subjective attributes. Q methodology assumes intra-individual differences in meaning, so that differences in views among participants. Thus, Q focuses on the relationship between a variable and a stimulus. In addition, factor analysis performed in parallel for this purpose is generally a method of classifying variables. In the R methodology, the variable becomes a test item or characteristic, but in the Q methodology, the respondent himself models it by comparing and ordering stimuli (usually statements), and eventually expresses his or her own opinion [21].

### Study procedure

#### Composition of concourse and Q-sample

In this study, a literature review on disaster and in-depth interviews with the nursing students were conducted in order to form the concourse. In Q, the concourse represents a universe of subjective items related to the topic. A semi-structured questionnaire was administered in order to establish various subjective statements through in-depth interviews; subjects that could have different opinions were selected [21] and proceeded until the data was saturated. To obtain a concourse about disaster safety of nursing students, the subjects for an in-depth interview were randomly selected from a total of 30 first, second, and third-year nursing college students in their twenties residing in city C, and then individual in-depth interviews were conducted.

The in-depth interview on disaster awareness of nursing students was conducted for about two weeks from August 1, 2020 to August 15, 2020, and 86 of the subjective items (concourses) were extracted through the in-depth interview process. However, a Q sample is typically between 40 and 60 items [21].

Duplicate questions from the concourse were deleted, as each topic had a common meaning and value. In addition to the researcher, three nursing professors participated to readjust the sentences. Thirty items made up the Q-sample and were selected through the process of exchanging opinions several times and revising whether or not they were composed of statements that clearly reveal the subjectivity of the study participants (Table 1).

**Table 1:** Q statement

1. Disaster management is difficult due to the shortage of young adults due to aging	16. The individual’s luck in the event of a disaster is important.
2. Disaster management is difficult if the emergency contact and cooperation system among residents is insufficient.	17. It is possible to keep personal property in case of a disaster.
3. Disaster management is difficult due to lack of local government’s equipment, manpower, and budget support.	18. When the disaster holds resources, it is stockpiling in the home.
4. Disaster management is difficult due to the lack of evacuation sites and facilities.	19. I know evacuation tips or situations when many disasters occur.
5. Disaster management is difficult if there are insufficient fire stations, health centers, and hospitals.	20. National and municipal responsibilities are important for safety and disaster management.
6. Disaster safety education should be essential in the school curriculum.	21. Talking about a disaster in the media is helpful for disaster safety.
7. Disasters happen.	22. Disaster training, training is necessary.
8. Disaster can kill me too.	23. I have a habit of checking emergency exits and emergency routes when in any place.
9. Disasters occur frequently and are growing in scale.	24. I have an emergency contact network in case of a disaster.
10. Disaster acts as an impediment to the country’s economic development.	25. I can induce disaster evacuation to those around me in the event of a disaster.
11. The State strives to prevent disasters and protect the people from the dangers.	26. The responsibility of each citizen is important for safety and disaster management.
12. It is enough to educate and prepare in advance through disaster education.	27, In most safety accidents and disasters, it is ignorance to nurture further.
13. Disaster policy and legislation aid in disaster safety guidelines.	28. You can pay more taxes for disaster and safety management.
14. Insensitivity to safety can lead to more disasters.	29. Government disasters and safety accidents are the result of negligence and oversight by agencies.
15. Most safety accidents and disasters are the result of irresponsibility and greed.	30. Korea has an efficient administrative system and legal basis for disaster management.

#### P-set selection

Based on this, a convenience sample of a total of 30 people, including those who had already participated in the interview, were recruited to form the P-set. The purpose and procedure of the study were explained to the subjects who participated voluntarily, and informed consent was obtained. In addition, it was explained that the subject can withdraw his/her intention to participate in the study at any time if he/she does not want it, and there will be no disadvantages.

#### Q-sort

Q sample classification is a process in which a subject classifies statements derived from the normal distribution of the Q distribution table and assigns a score to each item. A normal distribution reflects participants’ observations of a very strong positive or negative, for which a limited number of items can be ranked at the poles of the distribution. At the same time, a relatively large number of suborders are ranked toward the center of the distribution based on their relative indifference [21–23].

**Fig. 1:**
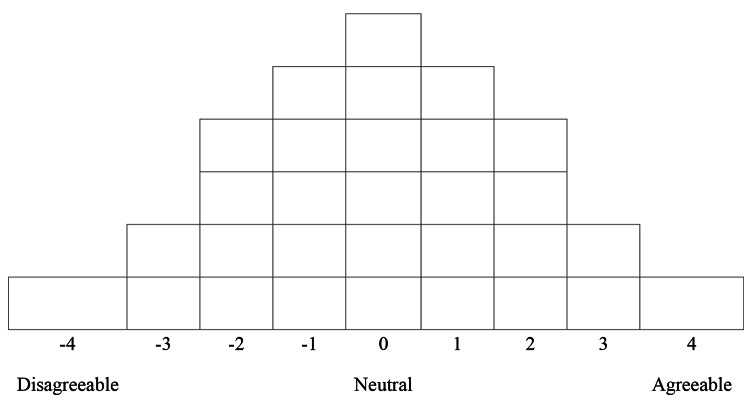
Card arrangement distribution table

The time required for Q-sorting, investigation on general characteristics, and interview was about 1 h. Data collection lasted about one month in September 2020. For the Q-sorting, Q-sample items were written in one statement on a paper card (Q-card) and numbered from 1 to 30 (the last number). Each statement card prepared in this way was read by the study participant and classified into three categories: agreement (+), neutral (0), and disagreement (-). After completing the classification into three groups, the statements of the Q-statement card group classified as agreement were re-read and re-classified in order from the strongest agreement (+ 4) to neutral (0) among the Q-statement cards. Similarly, the Q-statement card group classified as disagreement was classified in order from the strongest disagreement (-4) to neutral (0), and arranged according to the card arrangement distribution table shown in Fig. 1. After the classification was completed, we tried to obtain useful information for interpreting the Q-factors and gaining a broader understanding of the phenomenon through questions related to the reason or feeling of classification, and additionally disaster regarding the statements placed on both ends. The time required for the Q-sorting, questionnaire preparation, and interview was about 30 min to 1 h, on average, about 40 min. The study was carried out from June to December, 2020.

### Data analysis

This study was analyzed by using the principal component extraction with varimax rotation analysis within the PQ Method program (GNU general public license). The collected data were entered by assigning a point to the 30 Q-statements starting from − 4 for the strongest disagreement item to 0 for the neutral item, and 4 for the strongest agreement item depending on the level of agreement or disagreement for each participant’s Q-sort. The analysis in the PQ method is based on correlation and factor analysis. The determination of the number of factors is based on operancty. In practice, there are many cases where there is a difference between the statistical standard based on Eigen value 1 and the theoretical number of factors. It is more desirable to consider conceptual meaning and theoretical aspects rather than relying only on statistical criteria [23]. In this study, Q factor analysis was conducted for typing by applying principal component analysis and Varimax rotation. Then, identify the eigen value and cumulative variance of each factor and select them as appropriate factors. We confirmed that they were distinguished and identified detailed characteristics of each type. After identifying the characteristics of each type, the type name is named and interpreted. When interpreting the type, questions with an absolute standard score (Z-score) of 1.0 or higher are used to interpret the meaning of agreement and disagreement and determine the name of the type [24].

### Ethical considerations

The study was approved by the Institutional Research Board of K University (KHSIRB-19-012). The purpose and procedure of the study were explained to the subjects who voluntarily participated, and consent was obtained for the study. In addition, it was explained to the subjects that they could withdraw their intention to participate in the study at any time if they did not want to take part in the study, and that there would be no disadvantages.

## Results

### Result analysis

Among the 30 subjects in this study, during the classification process, 3 P samples (P-19, P-25, and P-27) that could not be explained by type characteristics as two or more factors were classified into complex types were excluded from the factor analysis. In addition, 26 out of 27 subjects were female and had experience in disaster education, whereas 1 male subject had no experience in disaster education. The age of the subjects ranged from 21 to 26 years old. In regard to religion, 2 subjects are Buddhists, 2 subjects are Catholics, 4 subjects are Protestants, and 19 subjects are non-religious. Those with experience of disasters had experienced typhoons, earthquakes, and floods. The subjects also said that disaster education was necessary in university lectures, public service advertisements, formal education, field education, and private education (Table 2).

**Table 2:** General characteristics and factor weights of P samples by type

Type	ID	Factor weights	Gender	Age	Disaster education	Religion	Disaster experience and type	Desired type of disaster education
Type1	P-6	0.52	F	26	Yes	None	Typhoon	③
(n = 13)	P-7	0.60	F	21	Yes	None	None	②, ③, ⑤
	P-11	0.68	F	21	Yes	None	Typhoon	④
	P-12	0.84	F	21	Yes	None	Typhoon, Flood	②, ③
	P-13	0.88*	F	21	Yes	Buddhism	Typhoon, Flood, Earthquake	②, ③
	P-14	0.67	F	21	Yes	Presbyterian	Typhoon, Earthquake	②, ③, ④, ⑤
	P-15	0.50	F	21	Yes	Catholic	Typhoon, Flood and Earthquake	③, ④
	P-16	0.65	F	21	Yes	None	Typhoon, Earthquake	④, ⑤
	P-17	0.60	F	22	Yes	None	Typhoon, Earthquake	③, ④
	P-18	0.55	F	21	Yes	None	None	②, ③, ④
	P-21	0.60	F	21	Yes	None	Typhoon, Earthquake	②, ④
	P-22	0.71	F	21	Yes	Presbyterian	Typhoon	②, ③
	P-26	0.64	F	21	Yes	Presbyterian	Typhoon	②, ③
Type2	P-2	0.72	F	23	Yes	None	Typhoon	⑤
(n = 6)	P-10	0.47	F	21	Yes	None	Typhoon	③, ④
	P-20	0.74*	M	24	None	Presbyterian	None	③, ④
	P-24	0.70	F	23	Yes	None	None	③, ④
	P-28	0.62	F	21	Yes	Catholic	Typhoon	③, ④
	P-29	0.60	F	21	Yes	Buddhism	Typhoon	⑤
Type3	P-3	0.73*	F	23	Yes	None	Typhoon	⑤
(n = 2)	P-9	0.72	F	21	Yes	None	None	②
Type4	P-1	0.70	F	23	Yes	None	Earthquake	①, ③, ⑤
(n = 6)	P-4	0.57	F	23	Yes	None	None	①, ③
	P-5	0.63*	F	23	Yes	None	None	①, ③, ④, ⑤
	P-8	0.53	F	21	Yes	None	None	④, ⑤
	P-23	0.59	F	21	Yes	None	Earthquake	②, ③, ④
	P-30	0.62	F	22	Yes	None	Typhoon	③, ④

* Typical of types

① University lecture ② Public advertisement ③ Regular education

④ Field training ⑤ Private training

In this study, four types of disaster awareness of nursing students according to the characteristics of each type are as follows: Type 1 was ‘national responsibility type’ in 13 students, type 2 was ‘individual responsibility type’ in 6 students, type 3 was ‘preparedness-oriented type’ in 2 students, and type 4 was ‘education-oriented type’ in 6 students. The Eigenvalue of this type is shown in Tables 3, and the explanatory power was 61%. Moreover, in the correlation between types showing similarity by type, the correlation coefficient between type 2 and type 4 was the highest at r = 0.50, and the correlation coefficient between type 2 and type 3 was lower at r = 0.04 (Table 4). The reason Type 2 and Type 4 are similar is inferred because Korea’s disaster education is an elective subject, and the reason the correlation between Type 2 and Type 3 is low is that Type 3 refers to the preparation of infrastructure.

**Table 3:** Eigen value and variance by type

	Type1	Type2	Type3	Type4
Eigen values	10.80	2.76	2.44	2.19
Variance (%)	24	16	8	13
Cumulative (%)	24	40	48	61

**Table 4:** Correlations among the types

	Type1	Type2	Type3	Type4
Type1	1.00			
Type2	0.48	1.00		
Type3	0.13	0.04	1.00	
Type4	0.48	0.50	0.19	1.00

### Analysis of the types

#### Type 1: National responsibility type

Subjects of type 1 were identified with 13 out of 27 participants, and all were female. The age ranged from 21 to 26, and all had experience in disaster education. Religion was Buddhist 1, Catholic 1, Protestant 3, and non-religious 8 (Table 2). It was characteristic that the subjects of Type 1 recognized that the government’s role and responsibility played an important role in disaster safety awareness (Table 5). In particular, they thought that in order to protect the rights of the people, the country should expand facilities, manpower, and resources. However, individual disaster capabilities were somewhat insufficient. The most important statements as of type 1 are as follows (Table 2): “*Isn’t it even in the constitution? There is a country to protect me. However, looking at what the country is doing with coronavirus these days, I think, as a nursing student, there seems to be a shortage of medical personnel. In particular, there are not enough nurses, but I am upset because the country seems to talk only about the simple idea of lack of manpower (P = 13).*” In addition, other participants in Type 1 also made statements such as wanting to receive a fair price from the state as a tax-paying citizen. Based on this, type 1 with these characteristics was named ‘national responsibility type’ in this study.

**Table 5:** Z score of type

	Type1	Type2	Type3	Type4
1. Disaster management is difficult due to the shortage of young adults due to aging	-1.20			-1.37
3. Disaster management is difficult due to lack of local government’s equipment, manpower, and budget support.		-1.09		
4. Disaster management is difficult due to the lack of evacuation sites and facilities.	1.12		1.86	
5. Disaster management is difficult if there are insufficient fire stations, health centers, and hospitals.	1.85			
7. Disasters happen.	1.46	2.23	-1.24	
8. Disaster can kill me too.	1.87	1.40	-1.86	1.16
9. Disasters occur frequently and are growing in scale.		1.02		
11. The State strives to prevent disasters and protect the people from the dangers.	1.24		1.86	1.85
12. It is enough to educate and prepare in advance through disaster education.			1.51	
13. Disaster policy and legislation aid in disaster safety guidelines.		-1.11		
14. Insensitivity to safety can lead to more disasters.		1.22	1.55	
16. The individual’s luck in the event of a disaster is important.		-1.12	-1.56	-2.63
18. When the disaster holds resources, it is stockpiling in the home.			1.24	-1.58
20. National and municipal responsibilities are important for safety and disaster management.			-1.24	1.01
22. Disaster training, training is necessary.		1.49	1.68	
23. I have a habit of checking emergency exits and emergency routes when in any place.	-1.87			
24. I have an emergency contact network in case of a disaster.	-1.62	-1.52	-1.24	-1.30
25. I can induce disaster evacuation to those around me in the event of a disaster.	-1.50			
28. You can pay more taxes for disaster and safety management.	-1.21	-1.66		
29. Government disasters and safety accidents are the result of negligence and oversight by agencies.		-2.03		

Blanks and deleted statements were not significant

#### Type 2: individual responsibility type

Type 2 subjects were represented by 6 out of 27 participants, including 1 female and 1 male. The age ranged from 21 to 24 years, and 5 subjects had experience in disaster education, but P-20, which is typical of Type 2, had no experience in disaster education. Religion was 1 Buddhist, 1 Catholic, 1 Protestant, and 3 non-religious (Table 2). As a result of factor analysis, the subjects of type 2 recognized that personal responsibility and role were important in disaster safety. They said that regular education, personal conscience, morality, etc. are necessary to develop individual disaster safety capabilities, and that personal ability and regular training are important when surviving in a disaster (Table 5). However, even though most of them received disaster education, they recognized that the individual’s capabilities were still insufficient, which could lead to death. Looking at this in detail, the statements of P-20 with the representativeness, the most important statement as of type 2 are as follows (Table 2): “*When I see it all the time, it seems that the cause of the damage is due to the greed of a bad company or individual to save a penny. So I think regular training is necessary.*” Another participant of the same type stated that the disaster situation was worsening due to the prevalence of individualism, and that we should help each other and make concessions. Based on this, type 2 with these characteristics was named ‘individual responsibility type’ in this study.

#### Type 3: preparedness-oriented type

Type 3 subjects were 2 out of 27, female, 21 and 23 years old, and all were non-religious (Table 2). The type 3 subjects recognized that disaster requires preparedness. In particular, they said that it was necessary to stockpile supplies or resources in preparation for a disaster, and recognized that both individuals and the country need to reserve facilities, resources, and manpower for this purpose. They believed that even in the event of a disaster, they could survive with sufficient preparation (Table 5). Looking at this in detail, the statements of P-3 with the representativeness, the most important statement as of type 3 are as follows (Table 2): “*I am still young, so I can’t die blindly. I check every time this happens, but I also have a survival bag at home and boxes of masks.*” Other participants of the same type Also, disasters can strike unexpectedly and countries may not be prepared for them. Therefore, it was stated that it is necessary for individuals to prepare and check in advance, and to avoid confusion in preparation for various disaster situations lurking in Korean society. Based on this, type 3 with these characteristics was named ‘preparedness-oriented type’ in this study.

#### Type 4: education-oriented type

Type 4 subjects were 6 out of 27, all of whom were female, aged 21 to 23 years, and all were non-educated (Table 2). The type 4 subjects were aware of the importance of education in disaster. They recognized that they needed to receive disaster education and train regularly, and that education and training should be systematically formed and provided at the national or local government level. They said that if proper education and training were given, it would increase the survival rate during emergencies, and people should always be aware that life could be lost. However, they also said that disaster education in the nursing department curriculum was insufficient. The type 4 subjects said that disaster training should be given through various curriculums, such as university, elementary, middle, and high schools, and private level (Table 5). Looking at this in detail, the statements of P-5 with the representativeness, the most important statement as of type 2 are as follows (Table 2): “*I think that not only nursing education, but also disaster education, should be conducted regularly starting from childhood and become a regular course. The education I am receiving poorly at the moment is insufficient and forgotten over time*.” Another participant of the same type also stated that since disaster education is provided only to certain occupations, awareness improvement education or experiential education for all citizens should be provided, and a system should be prepared so that they can receive it consistently. Based on this, type 4 with these characteristics was named ‘education-oriented type’ in this study.

## Discussion

In the undergraduate course of nursing college, education is needed to develop the ability to cope with various situations encountered in the field by establishing a value system for the nursing profession with a sense of humanity and nursing ethics [25]. The study by Goniewicz et al. [26] also emphasized that disaster education and training is a key component, and that exercises, as well as training and graduate studies focused on disaster response, are an important aspect of education and training activities. International organizations such as WHO and ICN criticize the undergraduate nursing education curriculum, which is insufficient to prepare nursing graduates to participate in disaster relief despite the increasing number of disasters worldwide [27, 28]. Kim and Lidia [27] stated that nurses respond to disasters at the forefront and that undergraduate education is necessary to equip them with basic competencies and knowledge to respond quickly in case of disasters. It is important that education properly prepares nurses to act in difficult situations due to unexpected circumstances [26]. Through this study, it is expected that the concept of disaster perceived by nursing students will be explored and will serve as basic data to develop the disaster nursing competency of nursing students.

As a result of this study, all four types indicate that individuals lack disaster capacity, and recognize the need for disaster education. This means that nursing students are acutely feeling the need for disaster education as in the current situation of the COVID-19 pandemic. Nursing students are not ready to respond to a disaster situation, but showing their willingness to participate in disaster response will greatly contribute to the development of nursing science in the future.

Looking at the results of this study, Type 1 place importance on the responsibilities and roles of the country. In the event of a major disaster, the belief system of the people changes differently, which may change their reactions based on their surroundings, thereby suggesting that a change in the disaster management paradigm is necessary [6, 29]. This means that accountability is imposed on the government and stakeholders, and the needs of the people in an interdependent relationship should be reflected with the common goal of minimizing damage in the disaster management system [6, 29]. In this study, the nursing students said that the government should prepare a systematic education program with human and material resources and systems in order to cultivate disaster awareness and disaster nursing competency. Therefore, in line with the needs of the nursing students, the country should take the initiative to prepare various systems for nurturing nurses who will be at the forefront of disaster response.

In the results of this study, Type 2 recognized that personal responsibility, competence, and morality were important. A nurse’s competency refers to the ability to integrate nursing care knowledge, ethical knowledge, personal knowledge, and aesthetic knowledge [30]. Labrague, et al. [7] reported that disaster-related experiences and awareness influence the ability to perform disaster nursing. In this study, recognizing the nursing students’ personal competence as important is a positive part of disaster management, and this supports the previous studies that nursing students want to actively respond to a disaster [31]. According to a study by Eom and Hwang [32] those who directly suffered or experienced disasters understood it as personal responsibility. Therefore, in future research, it is necessary to analyze people with personal responsibility in depth. Taken together, it is thought that preparing a program to improve individual factors such as personality, morality, and self-resilience is one way to raise awareness of disaster safety and enhance disaster nursing capacity.

Type 3 recognized the importance of active preparation for survival. Type 3 places importance on the roles of both the government and individuals in disaster preparedness, which includes establishing plans, organization, equipment, education, training, and cooperation systems [33]. It also implies that there is a demand from the people to diversify political, social, and economic structures, and develop strategies to prepare for damage caused by disasters [34].

As a result of this study, Type 4 recognized education related to disasters as important. The National Student Nurses’ Association (NSNA) emphasizes that nursing students must prepare for disasters in their individual, family, and nursing education courses and emphasizes education for this purpose [7, 35]. This seems to be the reason why nursing students feel that disaster education is insufficient, even though most of them have answered in this study that they have received disaster education. Therefore, it is necessary to identify the educational needs of nursing students for disaster education and develop a systematic curriculum based on this [13]. The study by Jung et al. [36] and the study by Kang and Piao [37] confirmed that the nursing students’ competency for disaster nursing increased through the disaster education program, and a number of previous studies also reported that the ability and knowledge to perform disaster nursing improved after education. It can be said that disaster education is essential for the nursing students who will play a pivotal role in the future potential disaster site. Therefore, in Korea, efforts are currently being made to develop disaster nursing education content in the community [38]. Each year, millions of people are affected by major events such as disasters, and disaster nursing education can have positive consequences for those affected by disasters in the future. As such, the characteristics of the types of disaster awareness of nursing students identified in this study have confirmed once again that it is urgent to prepare a disaster education program that reflects the diverse needs of undergraduate students.

Based on the results of the present study, in the undergraduate course of the nursing college, it is necessary to establish a value system for the nursing profession with a sense of humanity and nursing ethics, and to cultivate the ability to cope with various situations to be faced in the field [25]. Disaster nursing should prepare for disasters affecting the entire community, and provide nursing care not only for individuals and families, but also for groups and the entire community. Also, systematic analysis and education on learning goals or concepts for disasters are necessary [38–40].

Therefore, it is expected that this study will recognize the importance of nurses in a disaster situation, explore the concept of disaster recognized by the nursing students first, and become the basic data for nurturing the disaster nursing competency of the nursing students. In addition, confirming the change in disaster safety awareness of nursing students through the prepared education will provide insight in countless crises.

The study results can be used for research and development of nursing education strategies that would be effective in cultivating nurses who could prepare for various disaster situations. Furthermore, it can be used as an institutional device to enhance the competency of the nursing students. In further studies, experimental studies need to be conducted to develop an effective nursing education strategies or program, and to prove its effectiveness.

### Limitations

This study had some limitations. Since this study was conducted on nursing students living in a city, it is necessary to be careful when generalizing [41, 42] the study results, and there is a limitation in extending the interpretation to nursing students all in South Korea. Therefore, it is necessary to repeat and expand the study in the future while taking into consideration the sampling of the subjects. Also, the environmental situation, such as the frequency of disasters in the environment of the city in which the research was carried out, might affect the results of this study.

## Conclusions

Millions of people are affected by major events such as disasters each year, and disaster nursing education can have positive outcomes for those affected by disasters in the future. As such, the characteristics of the type of disaster awareness of nursing students confirmed in this study result confirmed once again the urgent need to prepare a disaster education program that reflects the diverse needs of students in the undergraduate course. It is expected that this study can be used as an institutional device and basic data to meet the public’s expectations and enhance the competency of nursing students at a time when the importance of disaster awareness increases and the public’s demand and interest increase. Through this study, the following points are suggested. First, it is proposed to develop a disaster nursing education program considering the types derived through this study and to apply it usefully in nursing. Second, an in-depth study on the types derived from this study is needed. Third, in addition to national disasters such as the current COVID-19 pandemic, natural disasters have different regional variations, so it is suggested to conduct repeated research that supplements these points.

## Data Availability

The datasets generated and/or analyzed during the current study are not publicly available due no new data were created or analyzed in this study, but are available from the corresponding author on reasonable request.
